# Too young to pour: the global crisis of underage alcohol use

**DOI:** 10.3389/fpubh.2025.1598175

**Published:** 2025-07-07

**Authors:** Susanna Esposito, Beatrice Rita Campana, Alberto Argentiero, Marco Masetti, Valentina Fainardi, Nicola Principi

**Affiliations:** ^1^Pediatric Clinic, Department of Medicine and Surgery, University of Parma, Parma, Italy; ^2^Università degli Studi di Milano, Milan, Italy

**Keywords:** underage drinking, alcohol consumption, alcohol prevention strategies, alcohol-related, youth substance abuse

## Abstract

**Background:**

Underage alcohol consumption remains a critical global public health concern, contributing to a wide spectrum of short- and long-term health risks. Despite age-based legal restrictions, alcohol persists as the most commonly used psychoactive substance among minors, outpacing tobacco, cannabis, and other drugs. Early initiation of alcohol use is strongly associated with heightened risks of addiction, impaired brain development, mental health disorders, and engagement in high-risk behaviors such as unintentional injuries, violence, and academic underperformance. Most research has focused on adolescents, while data on younger children remain scarce. Moreover, methodological inconsistencies in defining and measuring alcohol use across countries complicate international comparisons and the evaluation of policy interventions.

**Methods:**

This narrative review synthesizes contemporary literature on the epidemiology, determinants, and consequences of underage alcohol use. It examines genetic predispositions, family dynamics, peer influence, socioeconomic context, mental health, and exposure to alcohol-related media and advertising. It also evaluates the effectiveness of intervention strategies, including parental engagement, school-based education, extracurricular activities, community-level regulation, and professional health services.

**Results:**

Evidence highlights significant variability in the prevalence of underage drinking across regions, influenced by cultural, legal, and socioeconomic factors. Parental modeling, permissive attitudes, and weakened family structures are major contributors, while peer pressure and media exposure further normalize early alcohol use. Although various prevention strategies have demonstrated short-term benefits (particularly those involving active parental involvement and skill-based school programs), long-term effectiveness is limited due to inconsistent implementation, lack of standardization, and inadequate policy enforcement. Community-level interventions, such as increasing the legal drinking age and conducting compliance checks, have shown measurable success, but are underutilized in many regions.

**Conclusion:**

Addressing underage drinking requires a coordinated, multifactorial strategy. Broader investment in early prevention, standardized assessment tools, and targeted research on younger populations is essential. Strengthening policy enforcement and cross-sector collaboration will be critical to mitigate this growing public health challenge.

## 1 Background

Although with differences among countries, alcohol consumption is a significant clinical and public health concern worldwide. It is associated with severe health risks, including liver damage, cancer development, cardiovascular complications, and neurological and mental health disorders. Additionally, alcohol use can lead to profound social, occupational, and relationship problems, often resulting in premature death (1). The World Health Organization (WHO) estimated that in 2019, alcohol-related deaths reached 2.6 million globally. Furthermore, approximately 400 million people lived with alcohol use disorders, with 209 million diagnosed as alcohol dependent (2).

Among alcohol drinkers, a substantial portion consists of school children and adolescents. Alcohol is the most widely used substance among these age groups, surpassing tobacco, nicotine vaping, and marijuana (3). While overall alcohol use is typically more prevalent among adults, studies indicate that adolescents and young adults may engage in riskier patterns of use, such as binge drinking, at comparable or higher rates than adults (4–7). According to the most recent WHO/Europe report, over half of surveyed 15-year-olds had already experimented with alcohol (8). In the United States, the 2023 National Survey on Drug Use and Health found that 12.6 million individuals aged 12 to 20 (33.1% of this age group) had consumed alcohol at least once in their lives (9). Additionally, approximately 4,000 people under the age of 21 died from excessive alcohol use (3).

These statistics are particularly alarming, given that most countries prohibit the sale, provision, or offering of alcoholic products to minors, with variations in the legal drinking age (10). Despite these legal restrictions, underage alcohol use remains widespread and poses severe health consequences distinct from those observed in adults. Children who engage in heavy drinking are at a significantly higher risk of developing alcohol dependency and substance abuse disorders later in life. Moreover, alcohol-related chronic conditions such as liver cirrhosis, cardiovascular diseases, and certain cancers manifest earlier in children than in adults (11, 12).

Additionally, mental health problems are significantly more common and severe among adolescents who consume alcohol. Adolescence is a crucial period for biopsychosocial development, and alcohol use during this stage can disrupt brain maturation, leading to cognitive impairments and learning difficulties. Furthermore, early alcohol consumption is linked to higher engagement in risky behaviors, including motor vehicle accidents, homicides, falls, burns, drownings, and suicides. Underage alcohol abuse also imposes a substantial burden on public resources by increasing healthcare expenditures, contributing to educational failures, and escalating the demand for mental health services (13).

Despite these concerns, the epidemiology of alcohol use in children, particularly the youngest, remains poorly understood. Additionally, the social and economic factors contributing to underage drinking among very young individuals are not well-documented. This precludes identification of the best measures capable of preventing and reduce underage alcohol drinking. Finally, targeted initiatives by health authorities to combat alcohol consumption by children and adolescents are relatively uncommon worldwide. A WHO report indicated that in 2017, approximately 50% of countries, including several high-income nations, lacked programs for the treatment of children with alcohol and substance use disorders (14). This paper aims to summarize the current knowledge on underage alcohol drinking in children and adolescents in order to favor the identification of the problems whose solution can reduce epidemiology and consequences of this significant pediatric problems.

## 2 Methods

This is a narrative review that intends to synthesize the existing knowledge on underage alcohol consumption, including epidemiological data, determinants of use, and available interventions. No primary data were collected; rather, the study relied on a comprehensive literature search of empirical research, governmental reports, and authoritative health organization publications. A literature search was conducted across PubMed, Scopus, and Web of Science using predefined keywords such as “underage drinking,” “adolescent alcohol use,” “youth alcohol consumption,” “alcohol-related harm in minors,” and “prevention programs for underage drinking.” The search covered publications from January 2000 to January 2025, with additional seminal and policy-relevant studies included irrespective of publication date. Titles and abstracts were screened independently by two reviewers to assess eligibility. Full-text articles were retrieved for studies that met the inclusion criteria or where eligibility was uncertain. Discrepancies were resolved by consensus.

Inclusion criteria prioritized peer-reviewed empirical studies, government reports, and publications by international health organizations (e.g., WHO, CDC) that reported on prevalence, determinants, or interventions related to underage alcohol use. Studies were appraised based on methodological rigor (e.g., study design, sample size, measurement validity), relevance to the research question, and geographical diversity. Particular emphasis was placed on studies offering cross-national comparisons or policy evaluations.

Statistical data were extracted from authoritative sources to ensure accuracy in prevalence estimates and health outcomes. Program and policy evaluations were assessed based on implementation fidelity, reported effectiveness, and long-term impact. Although this review is not a meta-analysis, it follows structured narrative synthesis principles to ensure methodological transparency and reproducibility.

## 3 Results

### 3.1 Epidemiology of alcohol use in younger children and adolescents

Numerous studies have documented that many children consume alcohol; however, the precise epidemiology of underage alcohol consumption across different social and economic contexts remains poorly defined. Significant variations exist among countries, even among those with similar general characteristics. In the United States, results from the National Survey on Drug Use and Health have indicated that in 2021, among people aged 12 to 20, 15.1% (5.9 million) were past month alcohol users. Estimates of binge alcohol use and heavy alcohol use in the past month in the same population were 8.3% (3.2 million) and 1.6% (613,000), respectively (15). Significant differences among states were reported. Vermont had the highest rate of underage alcohol users, with nearly 25% consuming alcohol and 14% binge-drinking in the month before the survey. Mississippi, the state with the lowest underage consumption, at 9.7% for use and 5.4% for binge drinking (16).

In the European Union (EU), a 2019 survey revealed that 37.4% of children aged 15–16 years reported engaging in at least one episode of heavy drinking, defined as consuming five or more drinks on one occasion within the past 30 days. Notably, prevalence rates varied among Nordic countries, with lower rates observed in Iceland (8%), Norway (16%), Sweden (20%), and Finland (22%), but very high rates in Denmark, where the percentage of heavy drinkers was the highest (59%) among all European nations. Rates above the European average were also observed in Germany, Austria, Slovakia, and Croatia, where over 45% of adolescents engaged in heavy drinking (17).

Strong differences among countries were also reported in a systematic review and meta-analysis of 141 studies conducted in sub-Saharan Africa between 2018 and 2024, revealing varying lifetime and 6-month prevalence rates of alcohol use among children aged 10–17 years. Lifetime prevalence ranged from 13.2% in Ghana to 71.0% in Benin, while 6-month prevalence ranged from 1.2% in Uganda to 80.6% in Zambia and 46.3% in Seychelles (18).

Studies conducted in Southern American nations confirm that although a notable number of children and adolescents consume alcohol in all the countries, significant variation in prevalence among different countries can be demonstrated. A survey carried out in 14 Latin American countries (19) revealed that the lifetime prevalence of alcohol use among adolescents aged 14–17 years was 39% in Bolivia and 78.2% in Uruguay. Uruguay and Bolivia also reported the highest and lowest prevalence of alcohol use in the last year (67 and 28%) and the last month (50 and 16%), respectively. In Argentina, the lifetime, last year, and last month prevalence of alcohol use (63, 53, and 42%, respectively) was comparable to Paraguay (60, 49, and 40%), Chile (68, 57, and 40%), and Brazil (69, 67, and 48%). Other studies in Brazil (20) and Mexico (21) reported similar prevalence rates. Several factors contribute to these differences, including national regulations regarding alcohol consumption, heterogeneous study populations, and variability of methods for measuring alcohol consumption and duration. These inconsistencies complicate comparisons across studies and may impede adolescent research and policy planning.

Strict alcohol regulations can effectively deter underage drinking. Research indicates that measures, such as setting a minimum purchase age, reducing the density of alcohol outlets, and limiting sale days and hours can significantly decrease alcohol consumption among minors. Conversely, when state monopolies are replaced with private liquor stores or a licensed system, significant issues may arise. A study conducted in Sweden revealed that the privatization of the Swedish alcohol retail market was associated with a higher density of outlets, longer hours or more days of sale, changes in price, and an increase in consumption (22). However, legal drinking age policies differ significantly among countries. Some nations, primarily those with majority Muslim populations, impose stricter prohibitions on alcohol for the entire population. Conversely, in most industrialized countries, the minimum legal drinking age is 18 years, while in the United States, it is 21. Exceptions that facilitate alcohol use are common; for example, in the United Kingdom, where the legal drinking age is 18 years, alcohol consumption is permitted for 16-17-year-olds during meals when accompanied by an adult (23).

Regarding the heterogeneity of study populations, it is important to note that most studies focus on adolescents. However, the definition of adolescence varies. Generally, studies include individuals aged 10–18 years, but some extend the age range to 9–24 years, thereby increasing the number of participants classified as adolescents (24). Additionally, studies evaluating the initiation of alcohol use sometimes include much younger children. For example, research conducted in Uganda (25) and Peru (26) included children as young as 5 years old. Since the risk of alcohol use among these younger children is low, the global prevalence of alcohol consumption in pediatrics is lower than that reported in studies that enroll only adolescents.

The definition of alcohol consumption also influences epidemiological study results. Various criteria are used, including frequency of alcohol use, drinking quantity, weekly drinking, binge drinking, drunkenness, and alcohol-related problems. A greater prevalence of alcohol use is observed when it is defined as the intake of any alcohol, even in minimal amounts, while stricter definitions that account for blood alcohol concentration yield lower prevalence rates (27).

Alcohol consumption patterns among children can differ by sex, race, ethnicity, and study population composition. A study conducted across 44 countries in Europe, Central Asia, and Canada during the 2021/2022 period found that, in some countries, alcohol consumption was slightly more prevalent among girls than boys (28). In the United States, multiple studies have reported that White youth generally exhibit higher lifetime and 30-day drinking prevalence compared to African American youth. However, their alcohol use rates are similar to or lower than those of Hispanic youth, who often report higher levels of alcohol consumption (29). Table 1 summarizes epidemiology of underage alcohol use across different regions.

**Table 1:** Epidemiology of underage alcohol use across different regions.

Region	Age group	Prevalence of alcohol use	Binge drinking prevalence	Key findings
United States	12–20 years	33.1% have consumed alcohol at least once (8)	4,000 underage deaths annually due to excessive alcohol use (9)	Highest underage alcohol use reported in California and Texas (14)
European Union	15–16 years	37.4% engaged in heavy drinking (15)	59% in Denmark (highest in Europe)	Nordic countries report lower rates (8–22%) compared to central Europe
Sub-Saharan Africa	10–17 years	13.2% (Ghana) to 71.0% (Benin) lifetime prevalence (16)	1.2% (Uganda) to 80.6% (Zambia) 6-month prevalence	Wide variations due to socioeconomic factors and legal enforcement
United Kingdom	16–17 years	Allowed to drink alcohol with a meal if accompanied by an adult (17)	No official estimate provided	Legal drinking age is 18, but exceptions exist for younger individuals

### 3.2 Factors favoring underage alcohol use in children

Several factors can contribute to underage alcohol drinking. In most cases they operate within a multilevel framework. This is particularly evident when personal traits, parental influence, and peer pressure are considered. Impulsiveness to drink can derive from the desire to fit in with peers especially when parental supervision and guidance are lacking and when social and environmental stressors such as family conflict or socioeconomic challenges occur (30). However, in the following sections the role exerted by the single causes in conditioning alcohol initiation and long-term alcohol consumption are detailed.

#### 3.2.1 Age of alcohol initiation

An earlier initiation of alcohol use is often linked to problematic drinking later in life, including binge drinking, alcohol dependence, and alcohol use disorder, which can result in unintentional injuries and suicide attempts. However, it remains unclear whether early drinking directly causes future alcohol issues or merely indicates other risk factors (31). Early drinking might lead to increased alcohol use later by influencing relationships, social status, and self-conception through associations with risk-taking peers and prolonged exposure to an addictive substance. Alternatively, adult alcohol problems could stem from other underlying risk factors (32–34). A recent study (35) enrolling a Swedish nationwide sample of 4,018 adolescents aged 15/16 years at baseline and 17/18 years at follow-up seems to support the direct role of early drinking as cause of later problems. In this study it was shown that early drinking onset predicts subsequent higher alcohol consumption in late adolescence. Adolescents who had an early drinking onset drank more after 2 years than their peers who started later, regardless of the presence of other potential explanatory factors, such as sensation-seeking, Impulsivity, aggressivity peer relationship problems, conduct problems, and hyperactivity.

#### 3.2.2 Parental influence

Family characteristics, including genetics, play a significant role in shaping children’s drinking habits. Studies indicate that alcohol use disorders have a heritability of 50–60% (36), with genes related to the dopamine system—such as DRD2—and alcohol metabolism (ADH1B, ALDH2) influencing susceptibility to alcoholism (37). However, beyond genetics, the family environment is a crucial factor.

Parents serve as primary role models, and their drinking behaviors directly impact their children. Children with parents who consume alcohol regularly or have permissive attitudes toward underage drinking are more likely to develop alcohol problems. Several examples support this conclusion. Donovan and Molina (5) studying a sample of 452 children aged 8 or 10 years among whom 39% had sipped or tasted alcohol reported that the majority of sipping occurred in family contexts either during dinners or as part of family celebrations. Pilatti et al. reported that early alcohol exposure was largely under parental supervision in family settings when parents or other relatives allowed them to drink or were aware of their children’s drinking (38). An Australian study found that 33% of adolescents who drank in the past week received alcohol from their parents (39).

Additionally, children in households with drinking parents face increased risks of domestic abuse, loneliness, depression, anxiety, anger issues, poor academic performance, and an inability to trust others, all of which can contribute to underage drinking and substance use (40, 41). The correlation between family-related mental health issues and alcohol use is well-documented. A study in England on 11-16-year-olds showed that 36% of children with mental disorders had used alcohol, compared to 22.7% of those without (42). Continuous alcohol use was also higher in children with mental health disorders (31.7% vs. 19.4%). Notably, research suggests that mothers play a more influential role than fathers in shaping children’s drinking behaviors (43). Conversely, children raised in families with strict rules against alcohol and clear communication about its dangers are less likely to engage in drinking (44).

Family structure further impacts alcohol consumption. Adolescents living with both biological parents are less likely to engage in heavy drinking than those in non-intact families, where weakened parent–child relationships and reduced parental involvement contribute to risky behaviors (45). The risk is highest for children living with a single father or neither biological parent rather than a single mother (46). Furthermore, family stressors—such as financial difficulties, parental conflicts, or work-related stress—can lead children to use alcohol as a coping mechanism (47).

#### 3.2.3 Peer pressure

In addition to parental influence, peer pressure significantly contributes to underage drinking. During adolescence, the need for social acceptance is significant, and adolescents’ behaviors are heavily influenced by social norms, which include observed or unspoken behaviors (descriptive norms) and attitudes considered prevalent and acceptable within a peer group (injunctive norms). Studies consistently show that peer alcohol consumption and expectations are among the strongest predictors of adolescent alcohol problems (48). The perception of peer behavior is crucial in this context. Adolescents feel a strong urge to conform to the values and interests of their groups to maintain peer similarity and distinctiveness from other groups. Perceived higher prevalence of peer drinking predicts increased risk of alcohol initiation and consumption (49–55). The perception of peers’ approvals and disapprovals is particularly influential. A study conducted by Pedersen et al. (56) examined the impact of descriptive and injunctive norms on adolescent drinking behavior. These authors found that injunctive norms had the most substantial effect. While descriptive norms were associated with alcohol use, injunctive norms correlated with all drinking outcomes, including the consequences. On the other hand, peer disapproval of alcohol use is accompanied by reduced use of alcoholic beverages.

The study by Lee et al. (51) involving 1,808 middle school students (aged 13–15 years) found that higher peer nomination at baseline correlated with increased drinking occasions among initially alcohol-naïve students (adjusted Incidence Rate Ratio [aIRR = 1.06]). Conversely, having peers who opposed alcohol consumption was associated with a lower frequency of drinking (aIRR = 0.59).

#### 3.2.4 Media and advertising

Media portrayals and advertising significantly influence children’s perceptions of alcohol. Although exposure has declined slightly in recent years, it remains a pressing concern. Early 2000s studies reported that 1 in 6 magazine advertisements and 1 in 14 tele-vision ads for alcohol targeted underage drinkers (57). In 2002, youth were exposed to 65% more cooler advertisements, 45% more beer ads, and 12% more spirits ads than adults (58). More recently, between 2017 and 2018, underage viewers were exposed 28.5 billion times to alcohol advertisements on cable TV, with 651 million (2.3%) of these exposures violating industry placement guidelines (59).

Alcohol advertising frequently glamorizes drinking, associating it with fun, relaxation, and social success. TV shows, movies, and music videos reinforce this narrative, further normalizing alcohol consumption among impressionable youth (60–62). Longitudinal studies have confirmed that youth exposure to alcohol advertising correlates with earlier alcohol initiation, higher consumption levels, and negative health outcomes (63, 64). Notably, brand-specific advertising exposure among underage youth has been linked to higher consumption rates of those brands. One study found that alcohol consumption prevalence increased from 0.3% (no advertising exposure) to 8.7% (high exposure) (65). Similar patterns were observed in magazine advertising studies.

#### 3.2.5 Personality traits and preexisting health problems

Personality traits can affect patterns of thinking, feeling, and behaving. High levels of specific traits may increase the risk of alcohol problems by making one more susceptible to alcohol’s effects, more likely to use alcohol for emotional regulation, and more prone to risky behavior. Impulsivity is often elevated in alcoholics and heavy drinkers (66). Although alcohol’s impact on brain areas related to behavioral control complicates determining the direction of association, impulsivity can per se play a role in conditioning development of underage alcohol consumption as it can be demonstrated in children at risk before they start using alcohol beverages (67).

Certain preexisting physical and mental health conditions increase the likelihood of underage alcohol use. Adolescents experiencing chronic pain or somatic issues are more likely to suffer from mental health disorders, which, in turn, elevate their risk of alcohol consumption (68).

A study conducted in Italy on 6,506 adolescents (aged 11, 13, and 15) found that somatic problems were associated with 24% increased risk of regular alcohol use (69). Similarly, research by Conway et al. found that adolescents with preexisting mental health disorders (such as anxiety or behavioral disorders) had significantly higher rates of alcohol (10.3%) and illicit drug (14.9%) use (70). Early mental health issues are strongly linked to earlier alcohol initiation and an increased risk of problematic alcohol consumption.

### 3.3 Interventions to reduce underage alcohol drinking

Preventing and reducing underage drinking requires a multifaceted approach involving families, schools, communities, and policymakers. However, before exploring which intervention can be effective according to the main causes of underage alcohol consumption, it seems essential to shift social norms that allow alcohol use by young people. It is relatively common that young people, their parents and others in their communities frequently have a perceived culture of acceptance of youth alcohol consumption that leads to early and long-term alcohol drinking. A community-based social norms message delivered within a social marketing framework has been found able to modify this culture and considered the basis for a significant reduction of the underage alcohol problems (71). In the following section, present knowledge regarding the different measures used to reduce alcohol consumption in children and adolescents are detailed.

#### 3.3.1 Enhancing parental involvement

One of the most effective ways to reduce underage alcohol consumption is through active parental involvement. Research indicates that parenting behaviors significantly mediate the effects of interventions on adolescent drinking habits. Parents should engage with their children, take an interest in their activities and friendships, and foster open communication. Unlike school-based interventions, parental guidance can be tailored to the child’s needs and sustained over a longer period (72).

Parent-based interventions generally follow two approaches: general parenting strategies, which focus on sensation-seeking, emotion regulation, decision-making, and adolescent development, and alcohol-specific strategies, which provide information on the risks of early alcohol use. Studies suggest that a combination of both approaches is the most effective (73, 74) as evidenced by a meta-analysis by Bo et al., analyzing 20 studies published before March 2017 (75). Further research shows that changes in parental attitudes as a result of intervention programs significantly enhance their effectiveness (76). Additionally, open alcohol-related discussions between parents and adolescents help reduce underage drinking by increasing awareness of its consequences (77).

#### 3.3.2 Education and awareness programs

School-based interventions are another key strategy for reducing underage alcohol use. These interventions are effective because they can reach a large number of students, influence attitudes toward alcohol use before drinking begins, and counteract negative peer pressure (78, 79).

Education and awareness programs may be universal, targeting all students, or specifically designed for at-risk youth. Their integration into school curricula or behavioral management programs provides structured guidance on the short-term and long-term effects of alcohol, as well as strategies to resist peer pressure. However, the effectiveness of these programs remains debated (80–88). A Cochrane systematic review of 53 studies up to July 2010 identified methodological limitations in many studies, preventing firm conclusions (83). Some programs showed no effect, while others worked only for specific subgroups, such as girls or adolescents with preexisting drinking habits.

The effectiveness of these interventions depends on their structure and delivery method. Interactive programs that develop interpersonal skills have been found to yield better results than traditional lecture-based approaches (89, 90). Generic programs that focus on psychosocial and developmental aspects of behavior tend to outperform alcohol-specific interventions. Among these, the Life Skills Training Program (91), the Un-plugged Program (92), and the Good Behavior Program (93) have demonstrated significant reductions in alcohol use. Programs that focus on preventing the onset of alcohol consumption, rather than reducing existing use, have also been found to be more effective, particularly when implemented before the seventh grade (94–97).

Although long-term effects remain unclear, an Australian study with 13-year-olds found that a seven-year follow-up revealed a significant reduction in alcohol-related harms among students who participated in either universal (OR = 0.04) or at-risk-specific (OR = 0.17) interventions compared to controls (98). Online and mobile interventions, using social media, SMS, and apps, are emerging as an alternative to traditional methods. A review of 18 studies from 2005 to 2017 found that nine interventions, particularly those using SMS messaging, resulted in decreased alcohol consumption (99). However, further research with larger and more diverse samples is needed before these methods can be widely implemented.

#### 3.3.3 Promoting healthy activities

Engaging adolescents in structured extracurricular activities such as sports, arts, music, and volunteering can serve as a protective factor against alcohol use by providing positive outlets, building self-esteem, and fostering supportive social networks (100). However, the relationship between physical activity and alcohol use remains inconsistent. Some studies report no correlation, while others show both positive and negative associations.

For example, a study of 13,318 adolescents found that 13% of boys and 8% of girls avoided binge drinking when they did not participate in team sports (101). Another study suggested that physical activity can aid short-term alcohol abstinence and relapse prevention, though the data on intervention effectiveness were limited (102). A recent global study analyzing data from 222,495 adolescents across 66 countries found that physical activity was associated with lower odds of alcohol use (OR: 0.74) and binge drinking (OR: 0.66) (103). Interestingly, adolescents with lower sitting time were more likely to consume alcohol (OR: 1.68,) than those with excessive sitting time, suggesting that physical activity alone is not a reliable deterrent and should be complemented with other preventive measures.

#### 3.3.4 Community strategies for preventing underage drinking

Many countries have implemented community-based interventions aimed at reducing underage drinking by raising awareness and implementing regulatory measures. The effectiveness of media campaigns remains debated, with mixed results on their impact (104, 105). However, certain environmental strategies have been proven to work, including increasing the legal drinking age, raising alcohol prices through taxation, limiting the availability of alcohol by controlling the number and location of outlets, conducting sobriety checkpoints, and enforcing alcohol compliance checks (106, 107).

Studies have shown that enhanced law enforcement, including quarterly compliance checks, can significantly reduce underage drinking. A study by the CDC reported a decline in student alcohol use from 49.8 to 39.9% and a reduction in binge drinking from 32.0 to 25.0% after the implementation of such measures (108). Research suggests that these policies work not just by limiting access to alcohol but by increasing the perceived risk of enforcement and legal consequences, which serves as a deterrent for underage drinkers (109).

#### 3.3.5 Seeking professional help

Although experimentation with alcohol before the legal age is not necessarily indicative of problematic use, some children with documented alcohol use may benefit from targeted prevention efforts. Counseling and therapy can help address underlying emotional or behavioral issues, while family therapy may be beneficial in resolving household conflicts that contribute to substance use.

Standardized screening tools have been developed to identify children at risk. The National Institute on Alcohol Abuse and Alcoholism (NIAAA), in collaboration with the American Academy of Pediatrics, has created a two-question screening guide for children aged 9 to 18 years (110). The first question assesses whether the child’s friends consume alcohol, which helps determine their susceptibility to peer pressure and provides an opportunity to discuss alcohol-related issues. The second question focuses on the child’s personal drinking habits, allowing physicians to understand if alcohol use has already begun, its frequency, and its extent.

These simple yet effective questions enable early identification of at-risk children and allow practitioners to reinforce education on alcohol use, conduct brief interventions, and implement targeted prevention strategies. Seeking professional help can provide adolescents with the necessary tools to develop healthier coping mechanisms, ultimately reducing the risk of long-term alcohol dependency.

## 4 Discussion

On the basis of available literature it can be stated that alcohol consumption continues to pose a substantial burden on global health systems and societies, affecting individuals across the lifespan, including children and adolescents. Despite growing recognition of the issue, underage drinking remains pervasive, particularly among adolescents, where it is often normalized through cultural attitudes, peer behaviors, and media exposure. While the health risks of alcohol use in adulthood are well-established, the impact of early initiation (especially during critical developmental periods) deserves far greater attention.

Most pediatric studies and public health strategies focus primarily on adolescents aged 12 and older, often overlooking younger children who may be exposed to alcohol in familial or community settings. This narrow age focus has led to a research gap that limits our understanding of when alcohol-related behaviors begin to form, how they evolve, and what preventive strategies are most effective during early childhood. Evidence shows that exposure to alcohol (whether through direct consumption, parental modeling, or advertising) can begin much earlier than previously assumed. Studies have documented alcohol use or experimentation in children as young as 5 years old in certain socio-cultural contexts, highlighting the urgency of early intervention.

One of the most significant barriers to progress in this area is the lack of standardization in the definitions and measurement tools used to assess underage drinking (111). Different countries (and even studies within the same country) employ varying criteria for what constitutes alcohol use, binge drinking, or alcohol-related harm. This variability leads to inconsistent prevalence estimates, hinders accurate cross-national comparisons, and complicates the evaluation of preventive interventions. To move forward, there is a critical need for globally recognized, age-appropriate metrics to define and monitor underage alcohol use.

Research has consistently identified a range of interrelated risk factors that influence alcohol consumption among youth. Family dynamics (particularly parental alcohol use, permissive attitudes, or lack of supervision) play a central role. Children with one or more parents who consume alcohol regularly or struggle with substance use disorders are at markedly higher risk of early initiation. Equally influential is peer pressure, which becomes increasingly relevant during adolescence as young people seek social acceptance and autonomy. Media and advertising also exert a powerful effect, often glamorizing alcohol use and reinforcing false narratives about its role in social success, stress relief, or adult identity.

Additionally, the mental health dimension of underage alcohol use cannot be overstated. Numerous studies have shown a strong correlation between adolescent drinking and the presence of mood disorders, anxiety, behavioral problems, and experiences of trauma or neglect. Alcohol may serve as a coping mechanism for some youth, but its use can exacerbate underlying psychological issues, contributing to a dangerous feedback loop that increases the risk of long-term dependency and adverse life outcomes.

A wide array of prevention strategies has been developed, yet their implementation and impact remain uneven. Parent-focused interventions have shown considerable promise, particularly when they combine general parenting skills with alcohol-specific education. However, these programs often depend on voluntary participation and consistent parental engagement, which can be challenging to sustain. School-based programs are another vital component, especially those that begin in primary school and employ interactive, skills-based approaches rather than didactic lectures. Still, many of these initiatives are limited by insufficient funding, poor integration into the curriculum, or a lack of evaluation.

Community- and policy-level interventions (such as regulating alcohol availability, enforcing minimum legal drinking age laws, and conducting public awareness campaigns) have been associated with measurable reductions in underage drinking rates (Table 2). However, their effectiveness depends on enforcement strength, public buy-in, and sustained resource allocation. Moreover, digital media interventions, mobile health (mHealth) tools, and social media-based strategies represent emerging areas with significant potential, particularly for reaching tech-savvy youth, but more research is needed to assess their long-term impact.

**Table 2:** Key interventions for preventing underage alcohol use.

Intervention type	Description	Effectiveness	Challenges
Parental involvement	Programs targeting parenting behaviors, alcohol-specific discussions, and monitoring (51)	Effective when combined with general parenting strategies (g = −0.23) (54)	Requires parental commitment; effectiveness varies by family structure
School-based programs	Interactive interventions focusing on skill-building, awareness, and peer influence (57, 58)	More effective when initiated before grade 7; Life Skills Training, Unplugged, and Good Behavior programs show promising results (71–73)	Long-term effects uncertain; inconsistent implementation
Community strategies	Raising legal drinking age, alcohol taxation, limiting alcohol availability, enforcement measures (86, 87)	Effective in reducing underage drinking (CDC study: decline from 49.8 to 39.9%) (88)	Requires strong law enforcement and compliance checks
Professional help	Screening, counseling, and brief interventions for at-risk youth (89)	Early intervention reduces progression to alcohol dependency	Limited accessibility in many regions, stigma surrounding treatment

Despite these advances, underage alcohol use continues to evolve in response to social, cultural, and technological changes. Globalization, the growing influence of social media, and changing family dynamics all contribute to shifting risk profiles. As such, future research should prioritize adaptable, context-sensitive approaches that reflect the complex reality of young people’s lives. A coordinated, cross-disciplinary response is urgently needed to mitigate the long-term consequences of underage alcohol use. This includes not only more comprehensive surveillance systems and targeted interventions but also strong political will and intersectoral collaboration among healthcare providers, educators, policymakers, and community organizations. A particular focus should be placed on the early identification of at-risk children and the provision of tailored support before patterns of harmful alcohol use become entrenched.

## 5 Conclusion

Underage alcohol use is a multifaceted and persistent public health issue with serious short- and long-term consequences. Despite growing awareness and the development of numerous prevention strategies, progress remains hindered by research gaps, inconsistent methodologies, and variable policy enforcement. A unified, cross-sectoral approach is essential, one that emphasizes early detection, standardized assessment, and comprehensive intervention models tailored to both adolescents and younger children. Strategic investment in prevention, education, and policy will be key to reducing alcohol-related harm and fostering healthier outcomes for future generations.
